# NCHLT Auxiliary speech data for ASR technology development in South Africa

**DOI:** 10.1016/j.dib.2022.107860

**Published:** 2022-01-21

**Authors:** Jaco Badenhorst, Febe de Wet

**Affiliations:** a Voice Computing Research Group, CSIR Next Generation Enterprises and Institutions Cluster, P.O. Box 395, Pretoria 0001, South Africa; b Department of Electrical and Electronic Engineering, Stellenbosch University, Private Bag X1, Stellenbosch 7602, South Africa

**Keywords:** Speech data, Under-resourced languages, South African languages, Automatic speech recognition, Human language technology

## Abstract

The aim of the National Centre for Human Language Technology (NCHLT) project was to create speech and text resources that would enable Human Language Technology (HLT) development for the 11 official languages of South Africa. The speech data described in this paper was collected during the NCHLT project using a smartphone application. The official NCHLT Speech Corpus was released in 2014, but it did not include all recordings that were made during the data collection campaign. This paper describes the additional data that was recently released as auxiliary corpora [2]. The auxiliary data sets contain between 20 and 170 hours of speech data per language as well as the transcriptions associated with each utterance. In terms of the resources required for HLT development South Africa’s official languages are all under-resourced. The data described in this paper contributes toward alleviating this situation, specifically for the development of speech technology.

## Specifications Table

**Table:** 

Subject	Artificial intelligence/Computer Vision and Pattern Recognition/Applied Machine Learning/Signal Processing
Specific subject area	Automatic speech recognition
Type of data	Speech (audio)
	Transcriptions (text)
	Tables
How data were	Speech was recorded using a mobile application, *Woefzela*.
acquired	Participants read utterances displayed on a mobile device.
Data format	Raw
	Analysed (meta-data includes confidence scores for utterances)
Parameters for data collection	Prompts were recorded as separate utterances and saved in WAVE audio format. Multiple utterances were recorded per session (usually more than 200). Around 200 speakers of each language participated. Meta-data (e.g. recording location, age and gender) was captured during respondent registration and released with the data.
Description of data collection	Recording a representative sample of South Africa’s languages required a balance between rural and urban pronunciations. Data collection focused on one or two languages at a time in a best effort to capture speech diversity. However, since university campuses frequently served as collection sites the data might still be a non-representative sample of the general population’s speech. The language proficiency of respondents was verified by qualified language practitioners. A balance between male and female speakers was maintained and the majority of speakers were between 18 and 55 years old. Preceding the recordings, respondents participated in a training session to ensure the correct use of the mobile data collection application.
Data source location	Region: Africa
	Country: South Africa
Data accessibility	The NCHLT Auxiliary Speech data is available on a public repository.
	Repository Name:
	SADiLaR Language Resource Management Agency
	Direct URLs to data:
	Afr: https://hdl.handle.net/20.500.12185/522
	Eng: https://hdl.handle.net/20.500.12185/523
	Nbl: https://hdl.handle.net/20.500.12185/513
	Nso: https://hdl.handle.net/20.500.12185/518
	Sot: https://hdl.handle.net/20.500.12185/519
	Ssw: https://hdl.handle.net/20.500.12185/515
	Tsn: https://hdl.handle.net/20.500.12185/520
	Tso: https://hdl.handle.net/20.500.12185/521
	Ven: https://hdl.handle.net/20.500.12185/516
	Xho: https://hdl.handle.net/20.500.12185/514
	Zul: https://hdl.handle.net/20.500.12185/517
Related research article	Jaco Badenhorst & Febe de Wet, The usefulness of imperfect speech data for ASR development in low-resource languages, Information 2019, 10(9), 268.
	https://doi.org/10.3390/info10090268.

## Value of the Data

• The existing NCHLT Speech corpus only includes 50–60 hours of orthographically transcribed broadband speech per language [4]. The auxiliary data set contains more than double the number of hours for some of the languages in the corpus, all of which are under-resourced.
• The additional data will benefit speech technology advancement in South Africa since hundreds of hours of data are required to implement current state-of-the-art acoustic modelling techniques. This requirement remains a major challenge for the development of automatic speech recognition (ASR) systems and impedes further technological development for low-resource languages.
• The data contains additional examples of speech and provides more examples of languages recorded in varying acoustic conditions.
• It has been shown that combining this data set with existing speech data improves the recognition accuracy of ASR systems [2]. This is an important consideration, because there are currently almost no other resources available for speech technology development in South Africa. To participate in and engage with the digital future languages should be technologically enabled. Appropriate training data is a prerequisite to accomplish this aim.
• NCHLT resources have been applied in innovative use case scenarios. Speech-to-speech translation between South African languages was recently implemented in the AwezaMed mobile application. The app was developed to bridge and mitigate communication challenges in the health domain [6]. In a second use case, the NCHLT speech corpora were used to develop an automatic data harvesting procedure. The aim was to expand the speech resources available in South African languages by automatically transcribing data from the broadcast domain [3].

## 1 Data Description

The official NCHLT Speech corpus was released in 2014 and includes ±56 hours of data for each of South Africa’s 11 languages. The ±56 hour selections were made to meet the specifications stipulated by the funding agency that supported the project [4]. Data was selected such that the best quality data covering a balanced prompt set would be included in the official corpus (see Table 3 in [4]). Training (NCHLT_TRN) and test (NCHLT_TST) partitions of the official release were specified for each language.

**Table 1:** Aux2 speaker overlap for matching data fields (names (N), ID (I), and telephone numbers(T)).

Language	Mapped speaker identifier pairs	Mapped fields
Afr	006 650, 099 098, 166 161, 172 177, 506 164	ITN
	013 058, 505 127	IT, T

Eng	033 604, 097 699, 129 804, 134 806, 155 803, 172 801, 500 612, 617 805	ITN
	094 704, 095 632, 199 800, 617 805, 634 644, 650 658	IT
	127 802, 615 805, 638 805	TN, N, I

Nbl	002 614, 009 645, 010 639, 011 624, 018 640, 046 635, 618 657	ITN
	012 604, 014 606, 015 644, 023 659, 024 623, 024 632	IT
	016 629, 055 647, 093 649, 093 616, 093 656, 613 080, 626 653	T
	012 633, 064 658, 658 638	N
	013 627, 607 618, 026 617	TN, TN, I

Nso	102 648, 105 679, 162 667, 200 643	IN
	168 619, 195 613, 195 801, 613 801	T
	004 651, 115 802, 134 649	ITN
	076 695, 103 660, 116 700	TN, TN, IT
	179 614, 694 629, 072 680	N, N, I

Sot	072 670, 115 808, 138 805, 153 806, 187 801, 506 809	T
	807 660, 189 804, 179 803, 079 618, 097 668	ITN, IT, TN, I, N

Ssw	003 605, 004 619, 038 606, 041 617, 049 607, 046 604, 156 621, 160 613	IT
	014 618, 019 628, 025 601, 033 625, 039 629	ITN
	043 615, 051 623, 064 620, 188 616, 502 600	ITN
	013 608, 021 610, 035 626	I
	014 618, 017 624, 002 603, 155 609	IT, IT, TN, TN
	180 611, 618 622, 131 627	N, N, T

Tsn	036 673, 051 633, 051 640	T
	643 801, 113 802	ITN, TN

Tso	072 600, 113 606, 158 603	ITN
	139 605	IN

Ven	076 603, 090 660, 133 649, 155 675, 175 681	IT
	176 800, 180 667, 182 604, 187 643, 504 606	IT
	124 657, 137 654, 168 637, 190 633, 507 662	ITN
	117 636, 118 608, 139 674, 183 641, 159 802	I
	171 648, 618 650, 152 652, 022 609	IN, IN, TN, TN
	074 647, 122 672, 118 656	T, T, N

Xho	032 655, 070 615, 107 805, 134 800, 135 801	T
	167 804, 614 656, 622 636, 624 628	T
	010 692, 657 669	ITN
	014 695, 166 806, 600 656	N
	079 648, 183 802, 038 686	IT, TN, I

Zul	090 649, 181 802, 191 803, 192 801	T
	089 615	IN

The official corpus only includes a subset of the data collected during the NCHLT project. The additional data that was collected but not released before was made available in 2019 [2]. This data was gathered because the initial data collection process required adjustments for some languages. Earlier versions of the mobile application recorded prompts based on a prompt counter to assign the set of prompts to be recorded during a recording session. Unfortunately, field workers managing the recording devices sometimes cleared the memory where this value was stored by accident. As a result, some prompts were recorded multiple times while other prompts were never recorded. A second data collection campaign using an updated version of the software that included a random selection process to select more diverse prompt sets was therefore required.

To create the official NCHLT Speech corpus^1^ an NCHLT-baseline data set was first selected from all collected data. This initial pool of usable raw recordings included multiple sessions of some speakers and multiple examples of some prompts. The purpose of the NCHLT-baseline selection was to include recordings from the more diverse second collection effort, for cases in which some speakers participated in both data collection campaigns. The auxiliary (Aux) data constitute all recordings that are not part of the NCHLT Speech corpus. Two sets of Aux data have been defined as:

1. **Aux1**: The recordings left in NCHLT-baseline after selecting the data included in the official release.
2. **Aux2**: The recordings left in the pool of raw data after selecting NCHLT-baseline.

The auxiliary data is subdivided into 11 subsets, one for each language. A complete list of the language names and their corresponding ISO 639-3 codes are as follows: Afrikaans (Afr), South African English (Eng), isiNdebele (Nbl), isiXhosa (Xho), isiZulu (Zul), Sepedi (Nso), Sesotho (Sot), Setswana (Tsn), Siswati (Ssw), Tshivenda (Ven), and Xitsonga (Tso).

**Fig. 1:**
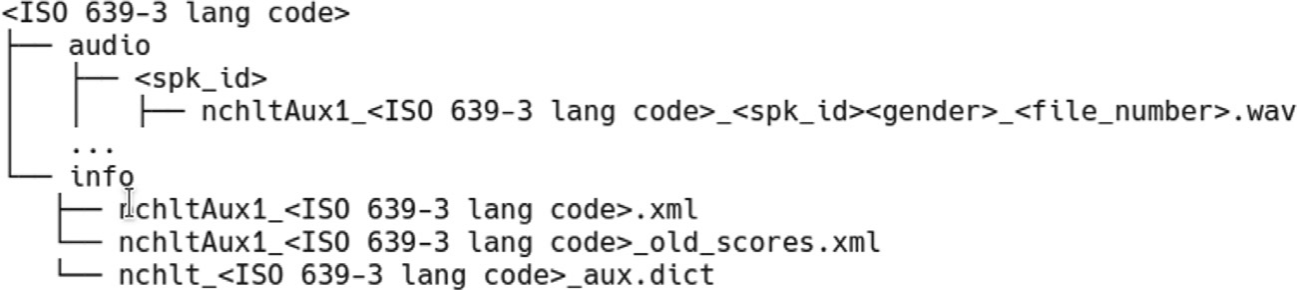
NCHLT Aux directory structure.

Individual recordings (utterances) were made in WAVE format files (16-bit, mono, PCM sampled at 16kHz) and labelled using a unique speaker identifier for every speaker. Fig. 1 shows the directory structure that was used to package the data for each language. The main directory consists of the relevant ISO 639-3 language code. It contains two sub-directories: the audio and info directories. The individual recordings were organised further according to speaker identifiers and the file naming convention included the above-mentioned language codes, the speaker identifiers concatenated with the characters “m” or “f” (indicating male or female speaker gender), as well as unique file numbers. Metadata on a per speaker and file basis as well as the prompt texts were captured in extensible markup language (XML) format. Each info directory contains two of these files as well as a file with pronunciations for all the words in the transcriptions (.dict). The old scores XML file in Fig. 1 includes phone-based dynamic programming (PDP) confidence scores [5] that were used to rank recordings in [4]. Confidence scores applied in [2] were included in the other file.

**Fig. 2:**
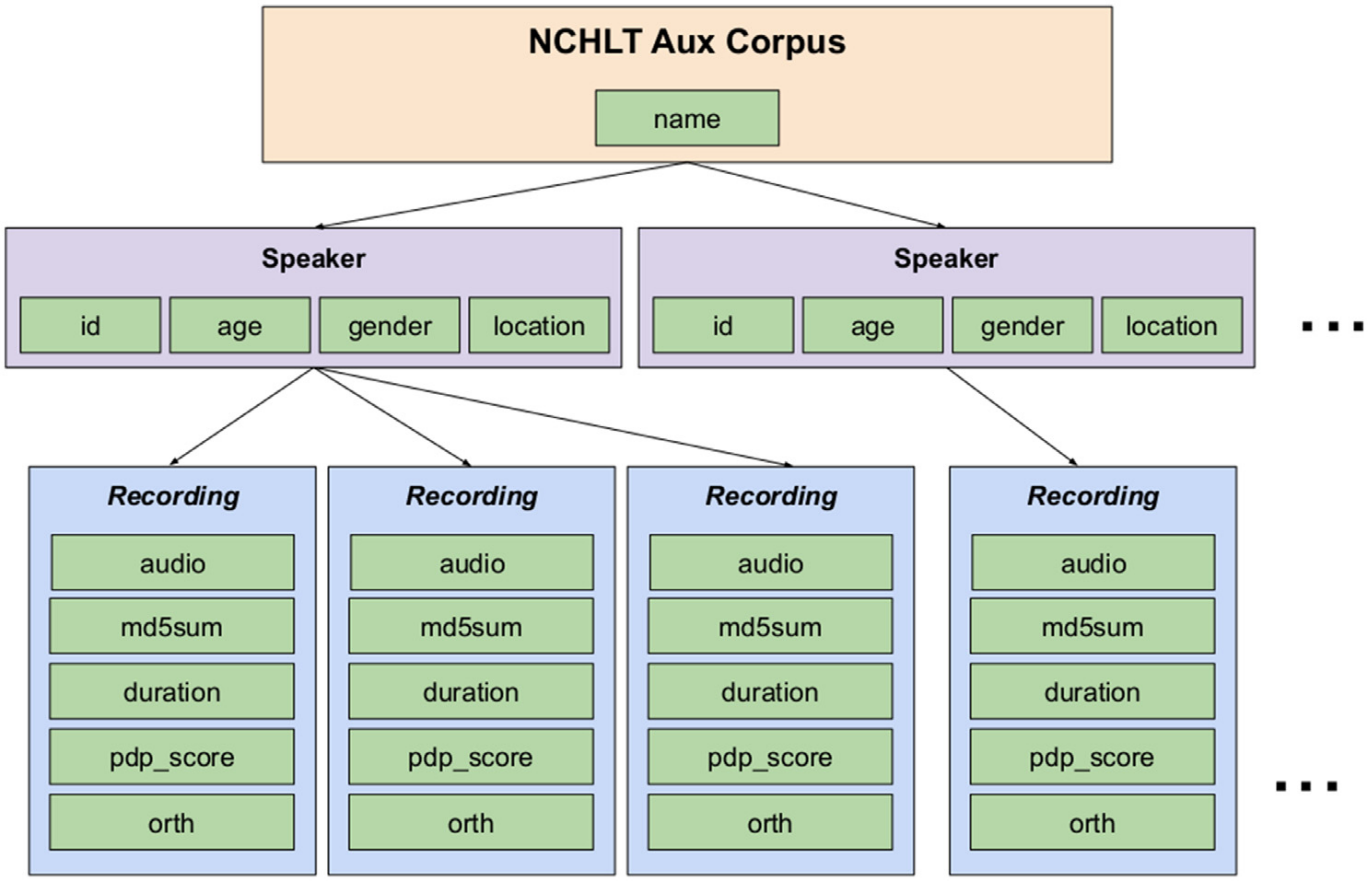
NCHLT Aux corpus XML definition.

Fig. 2 is a graphic representation of the XML metadata fields. The corpus definition has a hierarchy of three layers: corpus, speaker and recording. Each NCHLT Aux corpus comprises a single corpus layer, one or more speaker layers and many recording layers. Each layer implements metadata variables that are also applicable to lower layers in the hierarchy. At the Corpus level only the name variable of the corpus is defined, the name being one of the 11 ISO 639-3 language codes. All speaker layers are directly related to the single main corpus layer, but individual recordings are associated with particular speakers. The speaker layer variables include the anonymous numeric speaker identifier (id), followed by the age (a numeric value) and gender of the speaker: either the word male or female respectively. The majority of the speakers were in the age range between 18–55 and the ratio between male and female speakers is close to 50:50 for all the languages. A location variable captures the South African province where the speaker was recorded.

Recordings are relatively short segments of audio associated with a particular speaker only. The audio variable contains a link to an audio file in the form of a path. The md5sum and duration values corresponding to the audio file are included to enable consistency checks when the data is copied. The pdp_score field contains the confidence scores that were used to rank the recordings, while the orthography (orth) field contains the corresponding prompt text. In cases where the metadata failed basic checks or was not available, the corresponding field contains the value “-1”.

The audio file naming convention ensured unique file names across the entire corpus, which was required since including auxiliary NCHLT data means that duplicate speaker sessions could occur if the same respondent made multiple recordings. Aux1 speaker identifiers are the same as the original NCHLT identifiers since the selection of Aux1 was made from the NCHLT-baseline data set. No speaker overlap occurs between these two sets. For the Aux2 data, two speakers were mapped to the same speaker identifier according to criteria based on certain metadata fields and whether the contents of these fields were identical or not.

Table 1 lists speaker numbers that should pertain to the same speakers. A notation of speaker number pairs was used to associate any two identifiers with the same speaker identity in each language. Criteria used to determine the mapping between speaker identifiers included names (N), national identity (I) and telephone numbers (T). The name fields consisted of a character string including the typed name and surname of a respondent, the national identity was a sequence of 13 digits. Telephone numbers also consisted of digits. White space characters were ignored.

Since the meta data fields could contain typos and the speaker pairs derived from close matching (a difference of one character or digit only) fields provided a fair number of potential additional mappings, Table 2 was included. To anonymously convey which criteria was applicable to each speaker mapping, the mapped fields column lists the corresponding letters I, T or N as tags. Combinations of the letters such as ITN indicate that more than one criterion supported these speaker mappings. Some rows also contain lists of letter tags (separated by commas) where each tag corresponds to mapped speaker identifier pairs on a one-to-one basis.

**Table 2:** Aux2 speaker overlap for close matching data fields (names (N), ID (I), and telephone numbers(T)).

Language	Mapped speaker identifier pairs	Mapped fields
Eng	170 603, 127 802, 026 688	IT, I, T

Nbl	013 627, 093 656, 626 653, 627 636	I
	026 617	T
Nso	103 660, 502 652, 629 694	I
	162 667	T

Sot	138 805, 505 616, 644 807, 660 807	I

Ssw	014 618, 014 622, 155 609	I
	035 626, 180 611	T

Tso	139 605	T

Ven	022 609, 065 663, 118 656, 184 618	I
	118 608, 184 650, 618 650	IT
	139 674	T
Xho	600 656	I
	014 695	IT
	084 600, 166 806	T

Zul	054 622, 090 649, 180 647	I
	094 625	T

The number of speaker clusters created in this manner provides an indication of the extent of speaker overlap between the Aux2 and the other NCHLT corpora. The identifiers for speakers detected as the same person could subsequently be clustered together. Speaker clusters sometimes contained more than two speaker sessions. A summary of the speaker clusters was included in [2]. The number of speakers in the Aux2 data is much higher than the detected number of overlapping speakers. Therefore, Aux2 also contains data from additional speakers who are not represented in the NCHLT Speech corpus. In six languages, the Aux2 data also included a few speaker matches with the NCHLT_TST set. These speaker numbers can be located in Tables 1 and 2 as the numbers ranging between 500 and 599.

Table 3 provides an overview of the number of utterances (#Utt), the duration of the audio (Dur) and the number of speakers (#Spk) per language in each auxiliary corpus. The total duration of the Aux1 and Aux2 audio is 780.6 and 640.7 hours respectively.

**Table 3:** Total number of auxiliary (Aux1 and Aux2) utterances (#Utt) and corresponding duration (Dur) values (in hours) of additional data per language.

	Aux 1			Aux 2		
Lang	#Utt	Dur	#Spk	#Utt	Dur	#Spk
Afr	51 666	42.7	210	46 934	39.1	94
Eng	42 006	29.8	210	54 091	38.9	113
Nbl	34 445	42.6	148	96 200	120.1	208
Nso	62 965	64.9	210	52 371	51.8	105
Sot	68 599	73.9	210	47 238	43.5	98
Ssw	60 238	78.4	197	126 932	167.0	226
Tsn	67 702	70.2	210	34 800	37.0	75
Tso	67 530	83.7	198	827	0.7	6
Ven	78 009	93.7	208	43 446	54.9	86
Xho	81 821	103.0	209	50 720	55.0	107
Zul	74 362	97.9	210	29 992	32.7	63

**Total**	689 343	780.6	2220	583 551	640.7	1181

More information on the numbers of unique and repeated prompts and tokens in the NCHLT and NCHLT auxiliary corpora is provided in [2].

## 2 Experimental Design, Materials and Methods

Speech data was recorded during a dedicated data collection campaign using a smartphone application, *Woefzela*. The app was developed specifically for the purposes of the NCHLT project [7]. During the design of the Woefzela app, it was foreseen that remote recording environments would be much less controlled than for studio-based recordings. It was therefore decided to implement basic quality measures that could be performed on device and in semi-realtime. The aim was to ensure as many as possible successful respondent recording sessions. Successful recording sessions would contain enough recordings consisting of good speech samples.

At first, three basic quality checks were implemented: clipping detection, volume detection and speech cutting detection. While the aim of clipping detection was to identify whether microphone saturation occurred, both volume and speech cutting detection aimed to establish whether the recording contained valid speech. Finally a fourth speech sufficiency metric, aiming to detect adequate individual recording duration given the displayed text prompt, was included. The app automatically scheduled more recordings for sessions where some of the recordings failed the quality measures. In addition to the quality checks that were performed during data collection [1], PDP confidence scores for the individual utterances were verified before the corpora were compiled and released [5].

## Ethics Statement

The terms and conditions of the project were explained to all participants. Data was only collected from those who consented to their speech being recorded and included in the corpus. The criteria used to determine the mapping between speaker identifiers that included names, national identity and telephone numbers was removed prior to sharing and publishing the data. Only anonymised speaker identifiers were released with the data.

## CRediT authorship contribution statement

**Jaco Badenhorst:** Data curation, Writing – original draft. **Febe de Wet:** Supervision, Writing – review & editing.

## Declaration of Competing Interest

The authors declare that they have no known competing financial interests or personal relationships which have, or could be perceived to have, influenced the work reported in this article.

## Footnotes

1 In some publications related to the data this set is also referred to as the *NCHLT-clean* data.
